# Supplementary material for: Molecular crypsis by pathogenic fungi using human factor H. A numerical model
Source: PLoS One. 2019 Feb 19;14(2):e0212187. doi: 10.1371/journal.pone.0212187 (PMC6380567; doi:10.1371/journal.pone.0212187)
Supplement: S1 Fig — (PDF) [file pone.0212187.s001.pdf]

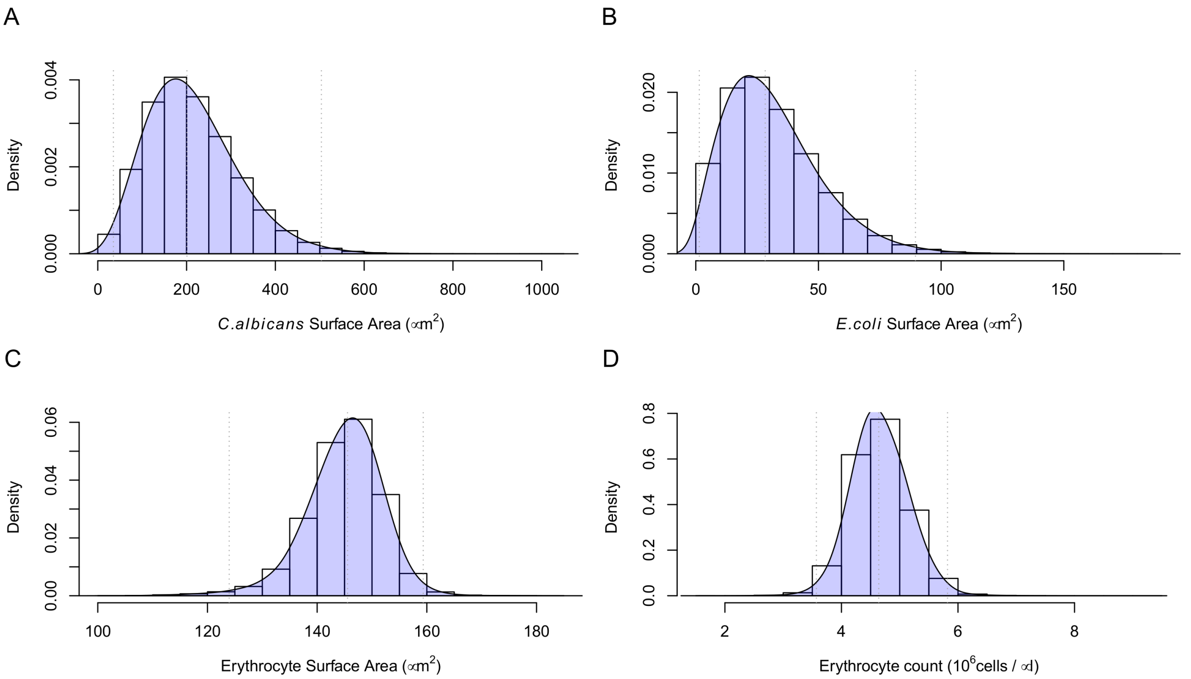

S1 Fig. Surface area distributions and erythrocyte count distribution used for sampling. Dotted vertical lines represent the 1st and 99th percentiles of the respective densities. (A) Density of *C. albicans* surface area assuming a spherical shape with a normally distributed diameter ($\mu = 8\,\mu m, \sigma = 2\,\mu m$). (B) Density of *E. coli* surface area assuming a spherical shape with a normally distributed diameter ($\mu = 3\,\mu m, \sigma = 1\,\mu m$). Erythrocyte surface area (C) and count (D) were estimated from the combined NHANES datasets from 2001 to 2014 [1]. The erythrocyte count is given directly in the data. To estimate erythrocyte surface area we used the mean cell volume and assumed a cylindrical geometry with a radius-to-height ratio of $\frac{7\mu m}{1.5\mu m} = 4.67$ (based on mean values in the literature).
