# Supplementary material for: Molecular crypsis by pathogenic fungi using human factor H. A numerical model
Source: PLoS One. 2019 Feb 19;14(2):e0212187. doi: 10.1371/journal.pone.0212187 (PMC6380567; doi:10.1371/journal.pone.0212187)
Supplement: S2 Fig — (PDF) [file pone.0212187.s002.pdf]

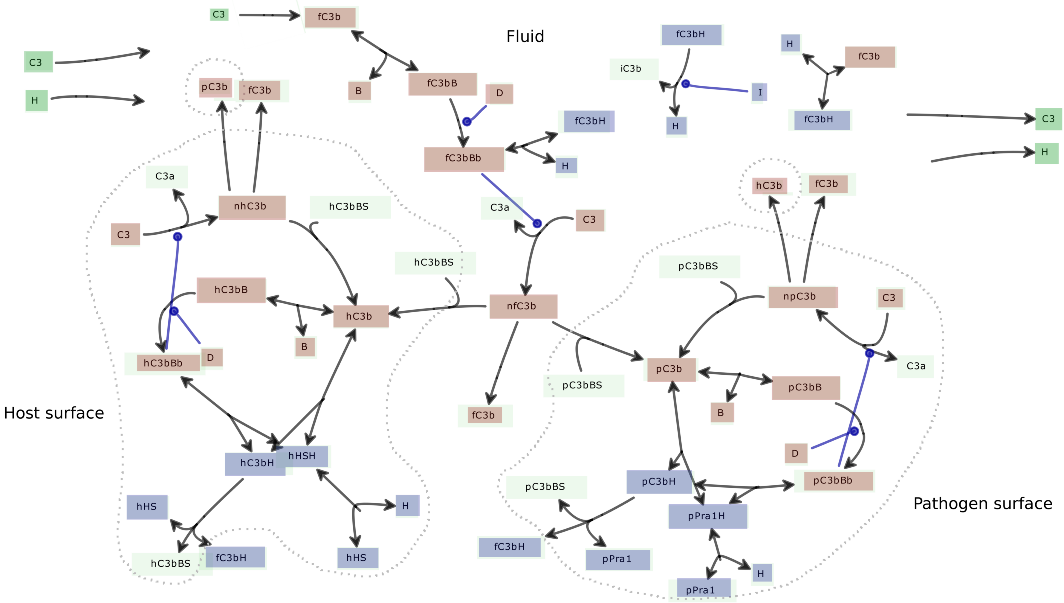

S2 Fig. Diagram of all reactions occurring in the *C. albicans* model. Inflow and outflow are indicated in green, amplification in red and regulation in blue. Starting with the tick-over reaction of C3 into fC3b (top-left), fC3b next associates in fluid with factor B to form the fluid C3 proconvertase (fC3bB). fC3bB is activated by factor D to form the active fluid C3 convertase (fC3bBb). fC3bBb may associate with factor H to form fC3bH, which is cleaved into iC3b by the fluid degradation reactions using factor I (top-right reactions, indicated in blue). If not inactivated, the active fluid C3 convertase (fC3bBb) may convert C3 into C3a and fluid nascent C3b (fnC3b). fnC3b may attach to either host or pathogen surfaces (see S2 Appendix), or associate with water to form fC3b (see S3 Appendix), which might start the fluid amplification loop again (if not regulated). If fnC3b hits a surface, the same reactions as in fluid may occur, eventually leading to the formation of the active C3 convertase bound to surfaces (pC3bBb and hC3bBb). See also Figs 1 and 3 in the main text for a schematic visualization of the reactions. The only differences in the reactions on surfaces and in fluid are that factor H may not attach to surfaces unless it is actively acquired by surface proteins (which are pPra1 for *C. albicans* and hHS for erythrocytes) and that a host or pathogen C3b binding site (hC3bBS and pC3bBS) is required for fnC3b to attach to surfaces.
