# Supplementary material for: Molecular crypsis by pathogenic fungi using human factor H. A numerical model
Source: PLoS One. 2019 Feb 19;14(2):e0212187. doi: 10.1371/journal.pone.0212187 (PMC6380567; doi:10.1371/journal.pone.0212187)
Supplement: S3 Fig — (PDF) [file pone.0212187.s003.pdf]

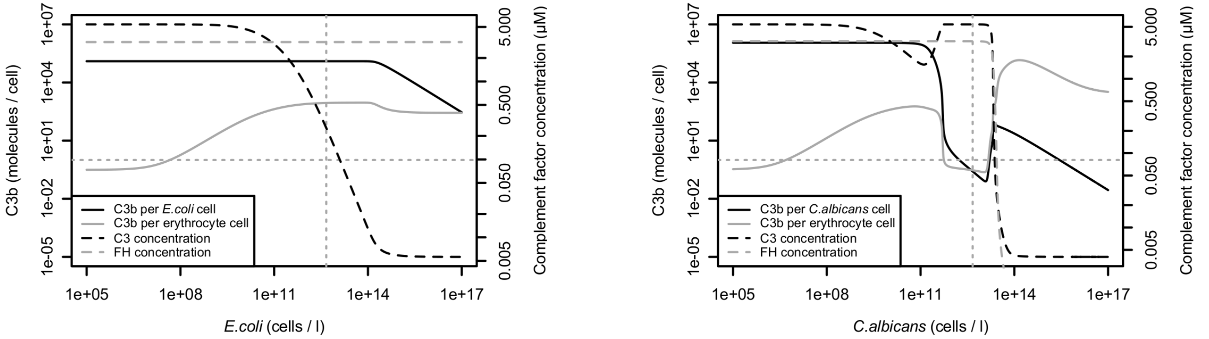

S3 Fig. Opsonization states and relevant complement factor concentrations assuming inflow of C3 and FH limited only by blood flow. Same data as used for creating the figures of the main text (Figs 6 and 8). Left, *C. albicans*: after the phase of successful crypsis, complement proteins FH and C3 decrease drastically. FH drops faster and the concentration is insufficient to protect both species. Since the binding affinity to FH is stronger on *C. albicans* surfaces, host opsonization increases faster. Right, *E. coli*: FH concentrations are not affected by increasing pathogen concentrations, but for high *E. coli* concentrations, C3 production is insufficient to ensure opsonization of both species.
