# Supplementary material for: Molecular crypsis by pathogenic fungi using human factor H. A numerical model
Source: PLoS One. 2019 Feb 19;14(2):e0212187. doi: 10.1371/journal.pone.0212187 (PMC6380567; doi:10.1371/journal.pone.0212187)
Supplement: S4 Fig — (PDF) [file pone.0212187.s004.pdf]

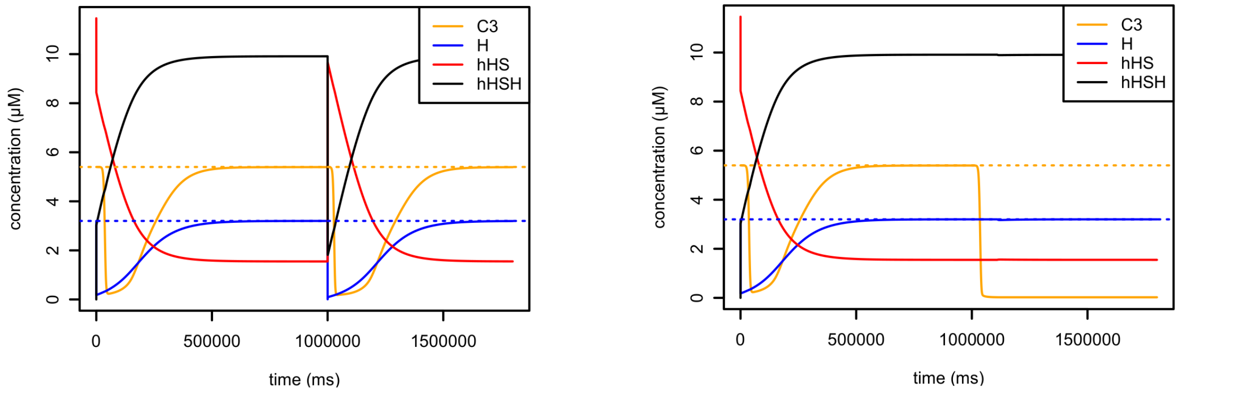

S4 Fig. Example dynamics assuming inflow of C3 and FH limited only by blood flow. Cell concentrations were chosen equally for host and pathogen. Left, *C. albicans*: directly after pathogen arrival at 1000 seconds, host cells loose a great deal of FH protection within a few milliseconds, in which the free heparan sulfate concentration (hHS) increases, and the bound heparan sulfate concentration (hHSH) decreases. After a period of about 250-500 sec, the original state is restored. This is because the binding of FH to pathogen surfaces is much stronger and dissociating FH from host surfaces gets sequestered by *C. albicans* immediately. Host protection recovers due to the inflow of FH. At even higher pathogen concentrations, FH inflow would not be sufficient to recover the protected state. In the case of *E. coli* (right) host protection is nearly unaffected, but C3 concentration decreases rapidly due to opsonization of the *E. coli* cells.
