# Supplementary material for: Molecular crypsis by pathogenic fungi using human factor H. A numerical model
Source: PLoS One. 2019 Feb 19;14(2):e0212187. doi: 10.1371/journal.pone.0212187 (PMC6380567; doi:10.1371/journal.pone.0212187)
Supplement: S6 Fig — (PDF) [file pone.0212187.s006.pdf]

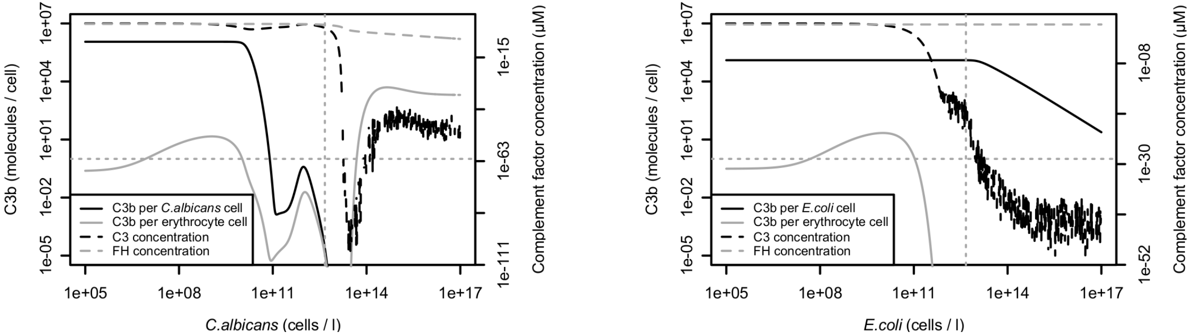


**S6 Fig. Opsonization states and relevant complement factor concentrations assuming no inflow of C3 and FH.** We see a faster drop in opsonization if molecular crypsis can be performed (left) but a short increase afterwards, before opsonization reaches zero. For high *C. albicans* concentrations, the pathogen remains without opsonization at all and only the host is opsonized. *E. coli* (right) opsonization is similar to the case with inflow, but the host has zero C3b bound on surfaces. Note that complement concentrations are very small for high pathogen concentrations and the numerical precision is not sufficient to perform accurate simulations.
