# Supplementary material for: Molecular crypsis by pathogenic fungi using human factor H. A numerical model
Source: PLoS One. 2019 Feb 19;14(2):e0212187. doi: 10.1371/journal.pone.0212187 (PMC6380567; doi:10.1371/journal.pone.0212187)
Supplement: S7 Fig — (PDF) [file pone.0212187.s007.pdf]

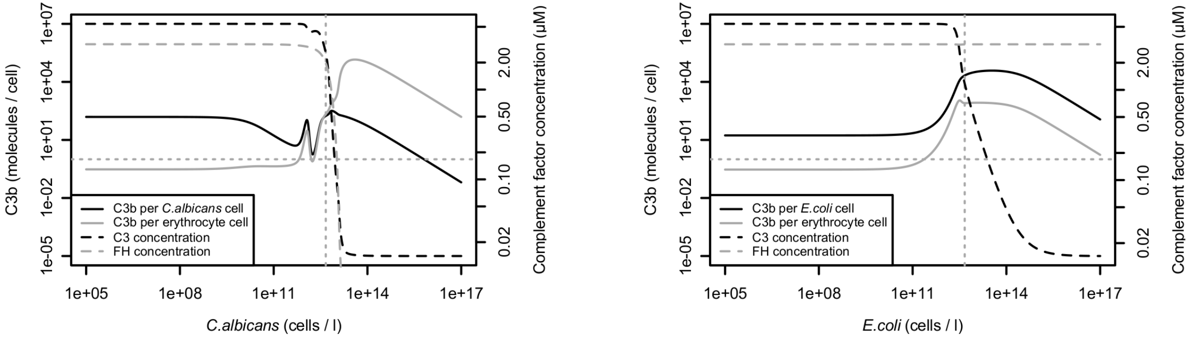


**S7 Fig. Opsonization states and relevant complement factor concentrations without the scaling factor accounting for spatial effects.** Without increased binding affinity of surface derived C3b to its originating surface, we see the same qualitative behaviour, but with less clear separability between host and pathogen cells. The regime where molecular crypsis is successful (left) is shorter and a oscillation of opsonization occurs. For *E. coli* (right) there could be a phase of inseparability even if the pathogen cells are less abundant than the host cells.
