# Supplementary material for: Molecular crypsis by pathogenic fungi using human factor H. A numerical model
Source: PLoS One. 2019 Feb 19;14(2):e0212187. doi: 10.1371/journal.pone.0212187 (PMC6380567; doi:10.1371/journal.pone.0212187)
Supplement: S8 Fig — (PDF) [file pone.0212187.s008.pdf]

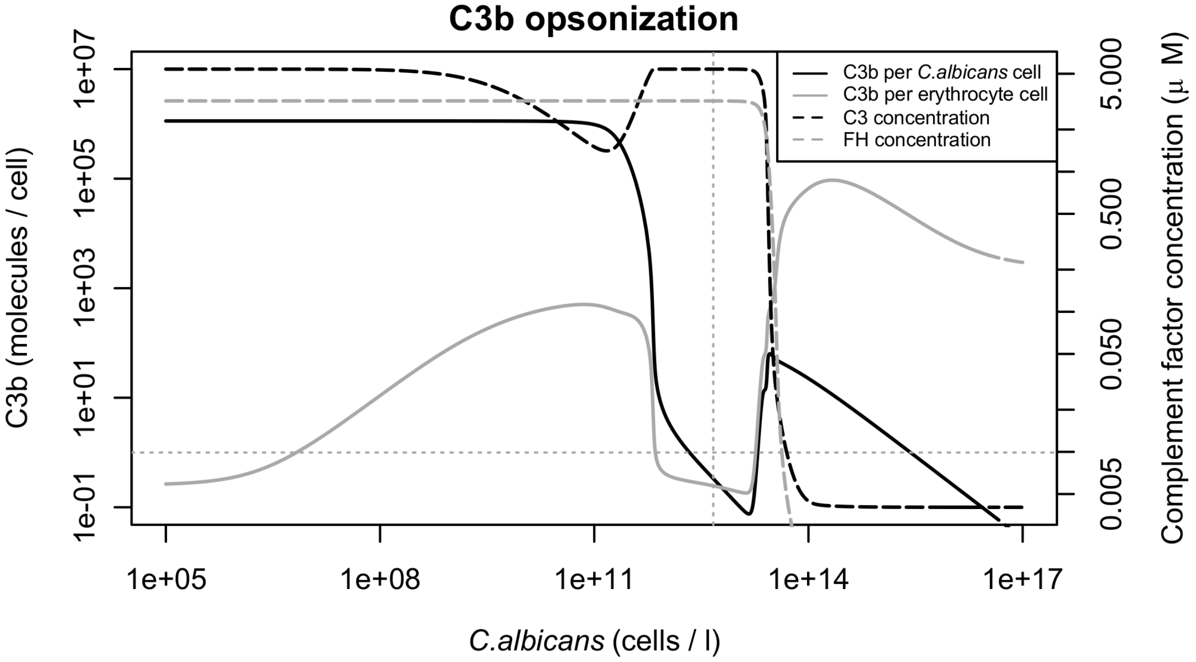

S8 Fig. Opsonization states and relevant complement factor concentrations with higher heparan sulfate concentration and lower Pra1 concentration on the surfaces. Heparan sulfate and Pra1 were increased and decreased, respectively, by 50 % compared to the standard values used. Variation in FH binding sites does not alter the dynamics in general, but a higher concentration of *C. albicans* cells compared to erythrocytes is needed to achieve the same qualitative behaviour as in Fig 6 and S3 Fig.
