# Supplementary material for: Molecular crypsis by pathogenic fungi using human factor H. A numerical model
Source: PLoS One. 2019 Feb 19;14(2):e0212187. doi: 10.1371/journal.pone.0212187 (PMC6380567; doi:10.1371/journal.pone.0212187)
Supplement: S9 Fig — (PDF) [file pone.0212187.s009.pdf]

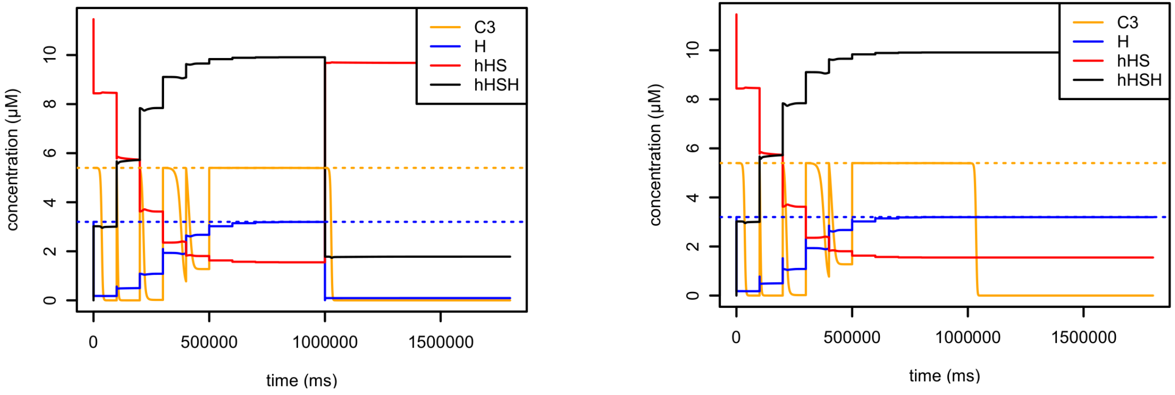

S9 Fig. Example dynamics assuming no inflow of C3 and FH. Cell concentrations were chosen equally for host and pathogen. Basically the same behaviour as in S4 Fig, but the host cannot recover its protected state and may be opsonized even for equal host and pathogen cell densities (at least if enough C3 is present).
