# Supplementary material for: Molecular crypsis by pathogenic fungi using human factor H. A numerical model
Source: PLoS One. 2019 Feb 19;14(2):e0212187. doi: 10.1371/journal.pone.0212187 (PMC6380567; doi:10.1371/journal.pone.0212187)
Supplement: S1 Table — (PDF) [file pone.0212187.s010.pdf]

S1 Table. Complement protein concentrations used in the model, as proposed by [2].

Complement proteins	Concentration (µM)	Source
C3	5.4	[8]
Factor H	3.2	[8]
Factor B	2.2	[8]
Factor D	0.083	[8]
Factor I	0.4	[8]
