# Supplementary material for: Molecular crypsis by pathogenic fungi using human factor H. A numerical model
Source: PLoS One. 2019 Feb 19;14(2):e0212187. doi: 10.1371/journal.pone.0212187 (PMC6380567; doi:10.1371/journal.pone.0212187)
Supplement: S2 Table — (PDF) [file pone.0212187.s011.pdf]

S2 Table. Kinetic rate constants used in the model, as proposed by [2].

Biochemical reaction	Rate Constant	Value	Source
Hydrolysis of $C3(H_2O)$	$k^{+}_{\mathrm{C3H_2O}}$	$8.3 \times 10^{-10}\,\mathrm{ms}^{-1}$	[9]
Association of factor B to C3b	k^{+}_{C3bB}	$0.000\,213\,\mathrm{\mu M}^{-1}\,\mathrm{ms}^{-1}$	[10]
Dissociation of complex C3bB	k^{-}_{C3bB}	$0.000\,155\,\mathrm{ms}^{-1}$	[10]
Attachment of fnC3b to host and pathogen	k^{+}_{fC3b}	$0.42\,\mathrm{\mu M}^{-1}\,\mathrm{ms}^{-1}$	Calculated, see S2 Appendix
Attachment of hnC3b to host	k^{+}_{hC3b}	varying	Calculated, see S5 Appendix
Attachment of pnC3b to pathogen	k^{+}_{pC3b}	varying	Calculated, see S5 Appendix
Association of nC3b (fnC3b, hnC3b and pnC3b) to water	k^{-}_{nC3b}	$11.55\,\mathrm{ms}^{-1}$	Calculated, see S3 Appendix
Association of factor H to C3b	k^{+}_{C3bH}	$0.0052\,\mathrm{\mu M}^{-1}\,\mathrm{ms}^{-1}$	[11]
Dissociation of complex C3bH	k^{-}_{C3bH}	$0.0325\,\mathrm{ms}^{-1}$	[11]
Association of factor H to heparin dp32/dp36 (HS)	k^{+}_{HSH}	$0.0065\,\mathrm{\mu M}^{-1}\,\mathrm{ms}^{-1}$	[12], estimated from dissociation constant
Dissociation of complex HSH	k^{-}_{HSH}	$0.003\,25\,\mathrm{ms}^{-1}$	[12], estimated from dissociation constant
Association of factor H to Pra1	k^{+}_{Pra1H}	$0.8673\,\mathrm{\mu M}^{-1}\,\mathrm{ms}^{-1}$	own measurements, estimated from dissociation constant
Dissociation of complex Pra1H	k^{-}_{Pra1H}	$0.001\,62\,\mathrm{ms}^{-1}$	own measurements, estimated from dissociation constant
Inflow of factor H	k^{+}_{H}	$4.88 \times 10^{-5}\,\mathrm{\mu M}\,\mathrm{ms}^{-1}$	Calculated, see S6 Appendix
Inflow of C3	k^{+}_{C3}	$8.23 \times 10^{-5}\,\mathrm{\mu M}\,\mathrm{ms}^{-1}$	Calculated, see S6 Appendix
Outflow of FH and C3	k^{-}_{blood}	$1.525 \times 10^{-5}\,\mathrm{ms}^{-1}$	Calculated, see S6 Appendix
Activation of complex C3bB by Factor D	k_{cat}C3bB<br>K_MC3bB	$0.0021\,\mathrm{ms}^{-1}$<br>$0.1\,\mathrm{\mu M}$	[2]
Cleavage of C3 by C3 convertase, C3bBb	k_{cat}C3bBb<br>K_MC3bBb	$0.0018\,\mathrm{ms}^{-1}$<br>$5.9\,\mathrm{\mu M}$	[13]
Cleavage of C3b by inhibitor Factor I	k_{cat}C3bH<br>K_MC3bH	$0.0013\,\mathrm{ms}^{-1}$<br>$0.25\,\mathrm{\mu M}$	[11]
