# Supplementary material for: Molecular crypsis by pathogenic fungi using human factor H. A numerical model
Source: PLoS One. 2019 Feb 19;14(2):e0212187. doi: 10.1371/journal.pone.0212187 (PMC6380567; doi:10.1371/journal.pone.0212187)
Supplement: S1 Appendix — (PDF) [file pone.0212187.s012.pdf]

S1 Appendix. C3b diffusion in the blood.
Taken from [2].

$$D_{\mathrm{C3b}} = \frac{k_B T}{6\pi\eta R} = 1.53 \cdot 10^{-11}\mathrm{m}^2\,\mathrm{s}^{-1}$$

Symbol	Value	Unit	Description
k_B	$1.38 \cdot 10^{-23}$	$\frac{\mathrm{kgm}^2}{\mathrm{s}^2\mathrm{K}}$	Boltzmann constant
T	310	K	temperature
η	0.004	$\frac{\mathrm{kg}}{\mathrm{m\,s}}$	viscosity of blood
R	$3.7 \cdot 10^{-9}$	m	C3b radius
