# Supplementary material for: Molecular crypsis by pathogenic fungi using human factor H. A numerical model
Source: PLoS One. 2019 Feb 19;14(2):e0212187. doi: 10.1371/journal.pone.0212187 (PMC6380567; doi:10.1371/journal.pone.0212187)
Supplement: S2 Appendix — (PDF) [file pone.0212187.s013.pdf]

**S2 Appendix. Binding rate of reactive C3b.**

After proteolytic cleavage, C3b occurs as a short-lived reactive intermediate, called "nascent" C3b (nC3b) [3]. nC3b is able to indiscriminately attach to different surfaces via an exposed internal thioester bond [4–6]. For reactive C3b, we assume a diffusion controlled reaction [7], where the binding reaction is assumed to occur spontaneously on contact with a cell surface.

$$k^{+}_{\mathrm{fC3b}} = 4\pi R D N_A = 4.2 \cdot 10^5 \frac{\mathrm{m}^3}{\mathrm{mol\,s}} = 4.2 \cdot 10^8 \mathrm{M}^{-1}\mathrm{s}^{-1}$$

| Symbol | Value | Unit | Description |
|---|---|---|---|
| $R$ | $3.7 \cdot 10^{-9}$ | m | C3b radius |
| $D_{\mathrm{C3b}}$ | $1.53 \cdot 10^{-11}$ | $\mathrm{m}^2\,\mathrm{s}^{-1}$ | diffusion coefficient C3b in blood |
| $N_A$ | $6.022 \cdot 10^{23}$ | | Avogadro's number |
