# Supplementary material for: Molecular crypsis by pathogenic fungi using human factor H. A numerical model
Source: PLoS One. 2019 Feb 19;14(2):e0212187. doi: 10.1371/journal.pone.0212187 (PMC6380567; doi:10.1371/journal.pone.0212187)
Supplement: S3 Appendix — (PDF) [file pone.0212187.s014.pdf]

S3 Appendix. Decay rate of reactive C3b.

Half live $t_{1/2}$ of about 60 µs for nC3b to remain active [3], before it reacts with water to form fluid-phase C3b (fC3b). In the model, this process is described with the reaction nC3b → fC3b and the following rate:

$$k^{-}_{\mathrm{nC3b}} = \frac{\ln 2}{t_{1/2}} = \frac{\ln 2}{60\mu\mathrm{s}} = \frac{\ln 2 \cdot 10^5}{6}\mathrm{s}^{-1} = 11550 \cdot \mathrm{s}^{-1}$$
