# Supplementary material for: Molecular crypsis by pathogenic fungi using human factor H. A numerical model
Source: PLoS One. 2019 Feb 19;14(2):e0212187. doi: 10.1371/journal.pone.0212187 (PMC6380567; doi:10.1371/journal.pone.0212187)
Supplement: S4 Appendix — (PDF) [file pone.0212187.s015.pdf]

S4 Appendix. C3b active hemispheric region.
With the half-time of C3b of

$$t_{1/2,C3b} = 60\mu\text{s},$$

we obtain the time after which 90 % of C3b have been inactivated:

$$t_{0.9,C3b} = 200\mu\text{s}.$$

So reactive C3b may diffuse a distance of

$$r_{\text{C3b}_{\text{active}}} = \sqrt{6D_{C3b}t_{0.9,C3b}} = 1356.3 \cdot 10^{-10}\text{m},$$

before 90 % of its activity is lost.
