# Supplementary material for: Molecular crypsis by pathogenic fungi using human factor H. A numerical model
Source: PLoS One. 2019 Feb 19;14(2):e0212187. doi: 10.1371/journal.pone.0212187 (PMC6380567; doi:10.1371/journal.pone.0212187)
Supplement: S5 Appendix — (PDF) [file pone.0212187.s016.pdf]

S5 Appendix. Scaling factor for the affinity of surface derived nascent C3b to the originating surface.

Adapted from [2].

Although there is no discrimination in binding affinity of nC3b to pathogen or host surfaces, the spatial location of the C3b amplification unit C3bBb (production side) has to be considered. The binding affinity k^{+}_{fC3b} of free C3b will only hold in a well mixed environment, namely for nfC3b produced in fluid by a fC3bBb amplification unit. For surface bound C3bBb units (host hC3bBb or pathogen pC3bBb) the local concentration of surface will be different than assumed in a homogeneous environment (where surface does not actually exist, but is assumed a homogeneous concentration of binding sites in the medium).

Since we know the radius of 90 % activity of C3b (S4 Appendix), we can neglect the non-planarity of the cell surface in this region and calculate the binding sites surrounding a single hC3bBb or pC3bBb unit as

$$N_{\text{local binding sites}} = \frac{A_{\mathrm{C3b}}}{\pi r_{\mathrm{C3b}_{\text{active}}}} = 578$$

where $A_{\mathrm{C3b}} = 100 \times 100\text{Å} = 10^{-16}\mathrm{m}^2$ is the surface area occupied by a single C3b molecule.

The local concentration of binding sites is then (assuming half spherical volume, because the membrane is impermeable to C3b)

$$c_{\text{local binding sites}} = \frac{N_{\text{local binding sites}}}{\frac{1}{2}V_{\mathrm{C3b}_{\text{active}}}N_A} = \frac{N_{\text{local binding sites}}}{\frac{2}{3}\pi r^3_{\mathrm{C3b}_{\text{active}}}\frac{1000\mathrm{l}}{\mathrm{m}^3}N_A} = 0.000\,183\,\mathrm{M}$$

where $N_A = 6.022 \cdot 10^{23}$ is Avogadro's number.

To account for this in the model, we could multiply the global binding site concentration $c_{\text{global binding sites}}$ of host and pathogen with the following factor:

$$s = \frac{c_{\text{local binding sites}}}{c_{\text{global binding sites}}}$$

But since the reaction is assumed to follow irreversible mass-action kinetics, we can equivalently multiply host and pathogen nC3b binding affinities with the same factor:

$$k^{+}_{\mathrm{hC3b}} = k^{+}_{\mathrm{fC3b}} \cdot s_h$$
$$k^{+}_{\mathrm{pC3b}} = k^{+}_{\mathrm{fC3b}} \cdot s_p$$

In summary this makes the binding affinity of nascent C3b to the originating surface higher than to other surfaces or fluid nascent C3b.
