# Supplementary material for: Molecular crypsis by pathogenic fungi using human factor H. A numerical model
Source: PLoS One. 2019 Feb 19;14(2):e0212187. doi: 10.1371/journal.pone.0212187 (PMC6380567; doi:10.1371/journal.pone.0212187)
Supplement: S6 Appendix — (PDF) [file pone.0212187.s017.pdf]

S6 Appendix. Inflow and outflow of relevant complement factors C3 and H in the blood stream.
Complement factors were first treated as external metabolites in the model. But for very high binding site concentrations this led to unrealistic scenarios where the amplification of C3b on pathogen surfaces (consuming C3) affected fluid phase amplification (also consuming C3) in a way that an explosion of concentrations occurred. Therefore we decided to model the inflow and outflow rates of the relevant complement factors H and C3 explicitly. Actual expression rates are hard to determine and may be extensively dependant on consumption rates due to gene regulation networks. Therefore we simplified this process by the idea that we have a static production site (e.g. the liver), where the concentration of complement factors is always kept constant (physiological conditions obtained from the literature, see S1 Table). It is only relevant then how often we pass this region based on mean blood-flow (cardiac output Q, blood volume V and the respective mean concentration of H_{global} and $\mathrm{C3}_{global}$ at the production site):

$$
\begin{aligned}
V_{blood} &= 5.25\,\mathrm{l} \\
Q &= 0.08\,\mathrm{l\,s^{-1}} \\
k^{-}_{blood} &= \frac{Q}{V} = 0.015\,25\,\mathrm{s^{-1}} \\
k^{+}_{H} &= k^{-}_{blood} \cdot \mathrm{H}_{global} = 4.88 \cdot 10^{-8}\,\frac{\mathrm{mol}}{\mathrm{l \cdot s}} \\
k^{+}_{C3} &= k^{-}_{blood} \cdot \mathrm{C3}_{global} = 8.23 \cdot 10^{-8}\,\frac{\mathrm{mol}}{\mathrm{l \cdot s}}
\end{aligned}
$$

With reactions:

$$
\begin{aligned}
&\mathrm{H} \xrightarrow{k^{-}_{blood}} \\
&\mathrm{C3} \xrightarrow{k^{-}_{blood}} \\
&\xrightarrow{k^{+}_{H}} \mathrm{H} \\
&\xrightarrow{k^{+}_{C3}} \mathrm{C3}
\end{aligned}
$$
