# Supplementary material for: Molecular crypsis by pathogenic fungi using human factor H. A numerical model
Source: PLoS One. 2019 Feb 19;14(2):e0212187. doi: 10.1371/journal.pone.0212187 (PMC6380567; doi:10.1371/journal.pone.0212187)
Supplement: S8 Appendix — (PDF) [file pone.0212187.s019.pdf]

References

1. Centers for Disease Control and Prevention (CDC) National Center for Health Statistics (NCHS). National Health and Nutrition Examination Survey Data. Hyattsville, MD: US Department of Health and Human Services, Centers for Disease Control and Prevention. 2001-2014;.

2. Zewde N, Gorham Jr RD, Dorado A, Morikis D. Quantitative modeling of the alternative pathway of the complement system. PloS one. 2016;11(3):e0152337.

3. Sim R, Twose T, Paterson D, Sim E. The covalent-binding reaction of complement component C3. Biochemical Journal. 1981;193(1):115–127.

4. Law S, Lichtenberg N, Levine R. Evidence for an ester linkage between the labile binding site of C3b and receptive surfaces. The Journal of Immunology. 1979;123(3):1388–1394.

5. Tack BF, Harrison RA, Janatova J, Thomas ML, Prahl JW. Evidence for presence of an internal thiolester bond in third component of human complement. Proceedings of the National Academy of Sciences. 1980;77(10):5764–5768.

6. Law S, Levine R. Interaction between the third complement protein and cell surface macromolecules. Proceedings of the National Academy of Sciences. 1977;74(7):2701–2705.

7. Collins FC, Kimball GE. Diffusion-controlled reaction rates. Journal of Colloid Science. 1949;4(4):425–437.

8. Zipfel PF, Skerka C. Complement: The Alternative Pathway. eLS. 2015;.

9. Pangburn M, Schreiber R, Müller-Eberhard H. Formation of the initial C3 convertase of the alternative complement pathway. Acquisition of C3b-like activities by spontaneous hydrolysis of the putative thioester in native C3. Journal of Experimental Medicine. 1981;154(3):856–867.

10. Chen H, Ricklin D, Hammel M, Garcia BL, McWhorter WJ, Sfyroera G, et al. Allosteric inhibition of complement function by a staphylococcal immune evasion protein. Proceedings of the National Academy of Sciences. 2010;107(41):17621–17626.

11. Pangburn MK, Mueller-Eberhard HJ. Kinetic and thermodynamic analysis of the control of C3b by the complement regulatory proteins factors H and I. Biochemistry. 1983;22(1):178–185.

12. Perkins SJ, Fung KW, Khan S. Molecular interactions between complement factor H and its heparin and heparan sulfate ligands. Frontiers in immunology. 2014;5.

13. Pangburn M, Müller-Eberhard H. The C3 convertase of the alternative pathway of human complement. Enzymic properties of the bimolecular proteinase. Biochemical Journal. 1986;235(3):723–730.
