# Supplementary material for: Molecular crypsis by pathogenic fungi using human factor H. A numerical model
Source: PLoS One. 2019 Feb 19;14(2):e0212187. doi: 10.1371/journal.pone.0212187 (PMC6380567; doi:10.1371/journal.pone.0212187)
Supplement: S5 Fig — (PDF) [file pone.0212187.s005.pdf]

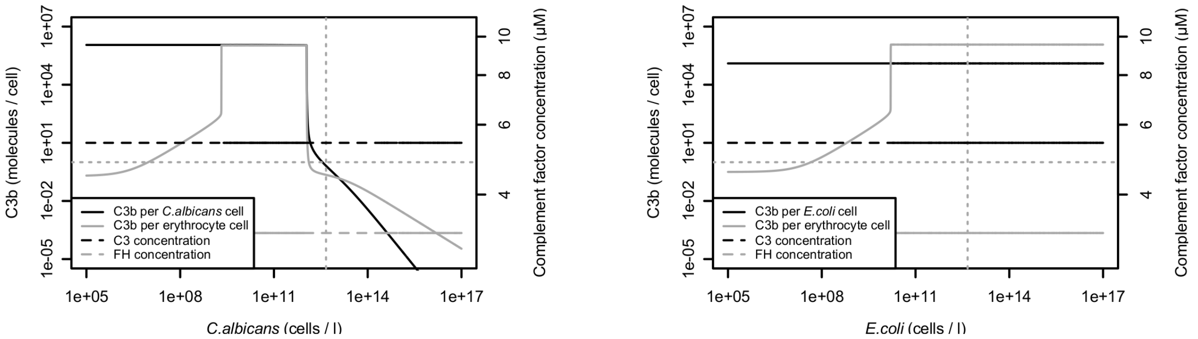

S5 Fig. Opsonization states and relevant complement factor concentrations assuming unlimited inflow of C3 and FH. We see rapid opsonization of both species even for low pathogen concentrations. This is due to the fact that the production of nascent C3b on surfaces influences fluid phase C3b amplification in a way that the regulation by FH is not sufficient to prevent amplification. Since C3 inflow is not limited, fluid phase nascent C3b explodes to infinite concentrations. A short decrease in C3 concentration (due to limited production / inflow) after complement activation seems necessary to prevent this. It is interesting to note nevertheless, that in the case of unlimited C3 and FH production, opsonization at high *C. albicans* (left) concentrations decreases for host and pathogen. This means that autoreactivity is unlikely to occur and local expression of FH, as it is done by for example epithelial cells, may help to prevent autoreactivity at least to some degree (expression must have certain limits).
