# Supplementary material for: Molecular crypsis by pathogenic fungi using human factor H. A numerical model
Source: PLoS One. 2019 Feb 19;14(2):e0212187. doi: 10.1371/journal.pone.0212187 (PMC6380567; doi:10.1371/journal.pone.0212187)
Supplement: S7 Appendix — (PDF) [file pone.0212187.s018.pdf]

S7 Appendix.

The complete ODE model.

$$\begin{aligned}\frac{\mathrm{d}\left([\mathrm{C3}]\cdot V_{\mathrm{Fluid}}\right)}{\mathrm{d}t} =\ & -V_{\mathrm{Fluid}}\cdot\left(\frac{\mathrm{kcat\_C3bBb}\cdot[\mathrm{pC3bBb}]\cdot[\mathrm{C3}]}{\mathrm{Km\_C3bBb}+[\mathrm{C3}]}\right)\\ & -V_{\mathrm{Fluid}}\cdot\left(\frac{\mathrm{kcat\_C3bBb}\cdot[\mathrm{hC3bBb}]\cdot[\mathrm{C3}]}{\mathrm{Km\_C3bBb}+[\mathrm{C3}]}\right)\\ & -V_{\mathrm{Fluid}}\cdot(\mathrm{kpC3H2O}\cdot[\mathrm{C3}])\\ & -V_{\mathrm{Fluid}}\cdot\left(\frac{\mathrm{kcat\_C3bBb}\cdot[\mathrm{fC3bBb}]\cdot[\mathrm{C3}]}{\mathrm{Km\_C3bBb}+[\mathrm{C3}]}\right)\\ & +V_{\mathrm{Fluid}}\cdot(\mathrm{kpC3})\\ & -V_{\mathrm{Fluid}}\cdot(\mathrm{kmFlow}\cdot[\mathrm{C3}])\end{aligned}$$

$$\begin{aligned}\frac{\mathrm{d}\left([\mathrm{H}]\cdot V_{\mathrm{Fluid}}\right)}{\mathrm{d}t} =\ & +V_{\mathrm{Fluid}}\cdot\left(\frac{\mathrm{kcat\_C3bH}\cdot[\mathrm{I}]\cdot[\mathrm{fC3bH}]}{\mathrm{Km\_C3bH}+[\mathrm{fC3bH}]}\right)\\ & -V_{\mathrm{Fluid}}\cdot((\mathrm{kpHSH}\cdot[\mathrm{hHS}]\cdot[\mathrm{H}]-\mathrm{kmHSH}\cdot[\mathrm{hHSH}]))\\ & -V_{\mathrm{Fluid}}\cdot((\mathrm{kpPra1H}\cdot[\mathrm{pPra1}]\cdot[\mathrm{H}]-\mathrm{kmPra1H}\cdot[\mathrm{pPra1H}]))\\ & +V_{\mathrm{Fluid}}\cdot(\mathrm{kpH})\\ & -V_{\mathrm{Fluid}}\cdot(\mathrm{kmFlow}\cdot[\mathrm{H}])\\ & -V_{\mathrm{Fluid}}\cdot((\mathrm{kpC3bH}\cdot[\mathrm{fC3b}]\cdot[\mathrm{H}]-\mathrm{kmC3bH}\cdot[\mathrm{fC3bH}]))\\ & -V_{\mathrm{Fluid}}\cdot((\mathrm{kpC3bH}\cdot[\mathrm{fC3bBb}]\cdot[\mathrm{H}]-\mathrm{kmC3bH}\cdot[\mathrm{fC3bH}]))\end{aligned}$$

$$\begin{aligned}\frac{\mathrm{d}\left([\mathrm{nfC3b}]\cdot V_{\mathrm{Fluid}}\right)}{\mathrm{d}t} =\ & -V_{\mathrm{Fluid}}\cdot(\mathrm{kmnfC3b}\cdot[\mathrm{nfC3b}])\\ & -V_{\mathrm{Fluid}}\cdot(\mathrm{kpfC3b}\cdot[\mathrm{nfC3b}]\cdot[\mathrm{hC3bBS}])\\ & -V_{\mathrm{Fluid}}\cdot(\mathrm{kpfC3b}\cdot[\mathrm{nfC3b}]\cdot[\mathrm{pC3bBS}])\\ & +V_{\mathrm{Fluid}}\cdot\left(\frac{\mathrm{kcat\_C3bBb}\cdot[\mathrm{fC3bBb}]\cdot[\mathrm{C3}]}{\mathrm{Km\_C3bBb}+[\mathrm{C3}]}\right)\end{aligned}$$

$$\begin{aligned}\frac{\mathrm{d}\left([\mathrm{fC3b}]\cdot V_{\mathrm{Fluid}}\right)}{\mathrm{d}t} =\ & +V_{\mathrm{Fluid}}\cdot(\mathrm{kmnfC3b}\cdot[\mathrm{nfC3b}])\\ & +V_{\mathrm{Fluid}}\cdot(\mathrm{kpC3H2O}\cdot[\mathrm{C3}])\\ & -V_{\mathrm{Fluid}}\cdot((\mathrm{kpC3bB}\cdot[\mathrm{fC3b}]\cdot[\mathrm{B}]-\mathrm{kmC3bB}\cdot[\mathrm{fC3bB}]))\\ & -V_{\mathrm{Fluid}}\cdot((\mathrm{kpC3bH}\cdot[\mathrm{fC3b}]\cdot[\mathrm{H}]-\mathrm{kmC3bH}\cdot[\mathrm{fC3bH}]))\\ & +V_{\mathrm{Fluid}}\cdot(\mathrm{kmnfC3b}\cdot[\mathrm{npC3b}])\\ & +V_{\mathrm{Fluid}}\cdot(\mathrm{kmnfC3b}\cdot[\mathrm{nhC3b}])\end{aligned}$$

$$\begin{aligned}\frac{\mathrm{d}\left([\mathrm{fC3bBb}]\cdot V_{\mathrm{Fluid}}\right)}{\mathrm{d}t} =\ & +V_{\mathrm{Fluid}}\cdot\left(\frac{\mathrm{kcat\_C3bB}\cdot[\mathrm{D}]\cdot[\mathrm{fC3bB}]}{\mathrm{Km\_C3bB}+[\mathrm{fC3bB}]}\right)\\ & -V_{\mathrm{Fluid}}\cdot((\mathrm{kpC3bH}\cdot[\mathrm{fC3bBb}]\cdot[\mathrm{H}]-\mathrm{kmC3bH}\cdot[\mathrm{fC3bH}]))\end{aligned}$$

$$\begin{aligned}\frac{\mathrm{d}\left([\mathrm{fC3bB}]\cdot V_{\mathrm{Fluid}}\right)}{\mathrm{d}t} =\ & +V_{\mathrm{Fluid}}\cdot((\mathrm{kpC3bB}\cdot[\mathrm{fC3b}]\cdot[\mathrm{B}]-\mathrm{kmC3bB}\cdot[\mathrm{fC3bB}]))\\ & -V_{\mathrm{Fluid}}\cdot\left(\frac{\mathrm{kcat\_C3bB}\cdot[\mathrm{D}]\cdot[\mathrm{fC3bB}]}{\mathrm{Km\_C3bB}+[\mathrm{fC3bB}]}\right)\end{aligned}$$

$$\begin{aligned}\frac{\mathrm{d}\left([\mathrm{fC3bH}]\cdot V_{\mathrm{Fluid}}\right)}{\mathrm{d}t} =\ & -V_{\mathrm{Fluid}}\cdot\left(\frac{\mathrm{kcat\_C3bH}\cdot[\mathrm{I}]\cdot[\mathrm{fC3bH}]}{\mathrm{Km\_C3bH}+[\mathrm{fC3bH}]}\right)\\ & +V_{\mathrm{Fluid}}\cdot(\mathrm{kpC3bH}\cdot[\mathrm{hC3bH}])\\ & +V_{\mathrm{Fluid}}\cdot(\mathrm{kpC3bH}\cdot[\mathrm{pC3bH}])\\ & +V_{\mathrm{Fluid}}\cdot((\mathrm{kpC3bH}\cdot[\mathrm{fC3b}]\cdot[\mathrm{H}]-\mathrm{kmC3bH}\cdot[\mathrm{fC3bH}]))\\ & +V_{\mathrm{Fluid}}\cdot((\mathrm{kpC3bH}\cdot[\mathrm{fC3bBb}]\cdot[\mathrm{H}]-\mathrm{kmC3bH}\cdot[\mathrm{fC3bH}]))\end{aligned}$$

$$\begin{aligned}\frac{\mathrm{d}\left([\mathrm{nhC3b}]\cdot V_{\mathrm{Fluid}}\right)}{\mathrm{d}t} =\ & -V_{\mathrm{Fluid}}\cdot(\mathrm{kphC3b}\cdot[\mathrm{nhC3b}]\cdot[\mathrm{hC3bBS}])\\ & +V_{\mathrm{Fluid}}\cdot\left(\frac{\mathrm{kcat\_C3bBb}\cdot[\mathrm{hC3bBb}]\cdot[\mathrm{C3}]}{\mathrm{Km\_C3bBb}+[\mathrm{C3}]}\right)\\ & -V_{\mathrm{Fluid}}\cdot(\mathrm{kmnfC3b}\cdot[\mathrm{nhC3b}])\end{aligned}$$

$$\begin{aligned}\frac{\mathrm{d}\left([\mathrm{npC3b}]\cdot V_{\mathrm{Fluid}}\right)}{\mathrm{d}t} =\ & -V_{\mathrm{Fluid}}\cdot(\mathrm{kppC3b}\cdot[\mathrm{npC3b}]\cdot[\mathrm{pC3bBS}])\\ & +V_{\mathrm{Fluid}}\cdot\left(\frac{\mathrm{kcat\_C3bBb}\cdot[\mathrm{pC3bBb}]\cdot[\mathrm{C3}]}{\mathrm{Km\_C3bBb}+[\mathrm{C3}]}\right)\\ & -V_{\mathrm{Fluid}}\cdot(\mathrm{kmnfC3b}\cdot[\mathrm{npC3b}])\end{aligned}$$

$$\begin{aligned}\frac{\mathrm{d}\left([\mathrm{hC3bBb}]\cdot V_{\mathrm{Fluid}}\right)}{\mathrm{d}t} =\ & +V_{\mathrm{Fluid}}\cdot\left(\frac{\mathrm{kcat\_C3bBb}\cdot[\mathrm{D}]\cdot[\mathrm{hC3bB}]}{\mathrm{Km\_C3bBb}+[\mathrm{hC3bB}]}\right)\\ & -V_{\mathrm{Fluid}}\cdot((\mathrm{kpC3bH}\cdot[\mathrm{hC3bBb}]\cdot[\mathrm{hHSH}]-\mathrm{kmC3bH}\cdot[\mathrm{hC3bH}]))\end{aligned}$$

$$\begin{aligned}\frac{\mathrm{d}\left([\mathrm{hC3b}]\cdot V_{\mathrm{Fluid}}\right)}{\mathrm{d}t} =\ & +V_{\mathrm{Fluid}}\cdot(\mathrm{kpfC3b}\cdot[\mathrm{nfC3b}]\cdot[\mathrm{hC3bBS}])\\ & +V_{\mathrm{Fluid}}\cdot(\mathrm{kphC3b}\cdot[\mathrm{nhC3b}]\cdot[\mathrm{hC3bBS}])\\ & -V_{\mathrm{Fluid}}\cdot((\mathrm{kpC3bB}\cdot[\mathrm{hC3b}]\cdot[\mathrm{B}]-\mathrm{kmC3bB}\cdot[\mathrm{hC3bB}]))\\ & -V_{\mathrm{Fluid}}\cdot((\mathrm{kpC3bH}\cdot[\mathrm{hC3b}]\cdot[\mathrm{hHSH}]-\mathrm{kmC3bH}\cdot[\mathrm{hC3bH}]))\end{aligned}$$

$$\begin{aligned}\frac{\mathrm{d}\left([\mathrm{hC3bB}]\cdot V_{\mathrm{Fluid}}\right)}{\mathrm{d}t} =\ & +V_{\mathrm{Fluid}}\cdot((\mathrm{kpC3bB}\cdot[\mathrm{hC3b}]\cdot[\mathrm{B}]-\mathrm{kmC3bB}\cdot[\mathrm{hC3bB}]))\\ & -V_{\mathrm{Fluid}}\cdot\left(\frac{\mathrm{kcat\_C3bBb}\cdot[\mathrm{D}]\cdot[\mathrm{hC3bB}]}{\mathrm{Km\_C3bBb}+[\mathrm{hC3bB}]}\right)\end{aligned}$$

$$\begin{aligned}\frac{\mathrm{d}\left([\mathrm{pC3b}]\cdot V_{\mathrm{Fluid}}\right)}{\mathrm{d}t} =\ & +V_{\mathrm{Fluid}}\cdot(\mathrm{kppC3b}\cdot[\mathrm{npC3b}]\cdot[\mathrm{pC3bBS}])\\ & -V_{\mathrm{Fluid}}\cdot((\mathrm{kpC3bB}\cdot[\mathrm{pC3b}]\cdot[\mathrm{B}]-\mathrm{kmC3bB}\cdot[\mathrm{pC3bB}]))\\ & +V_{\mathrm{Fluid}}\cdot(\mathrm{kpfC3b}\cdot[\mathrm{nfC3b}]\cdot[\mathrm{pC3bBS}])\\ & -V_{\mathrm{Fluid}}\cdot((\mathrm{kpC3bH}\cdot[\mathrm{pC3b}]\cdot[\mathrm{pPra1H}]-\mathrm{kmC3bH}\cdot[\mathrm{pC3bH}]))\end{aligned}$$

$$\begin{aligned}\frac{\mathrm{d}\left([\mathrm{pC3bBb}]\cdot V_{\mathrm{Fluid}}\right)}{\mathrm{d}t} =\ & +V_{\mathrm{Fluid}}\cdot\left(\frac{\mathrm{kcat\_C3bB}\cdot[\mathrm{D}]\cdot[\mathrm{pC3bB}]}{\mathrm{Km\_C3bB}+[\mathrm{pC3bB}]}\right)\\ & -V_{\mathrm{Fluid}}\cdot((\mathrm{kpC3bH}\cdot[\mathrm{pC3bBb}]\cdot[\mathrm{pPra1H}]-\mathrm{kmC3bH}\cdot[\mathrm{pC3bH}]))\end{aligned}$$

$$\begin{aligned}\frac{\mathrm{d}\left([\mathrm{pC3bB}]\cdot V_{\mathrm{Fluid}}\right)}{\mathrm{d}t} =\ & +V_{\mathrm{Fluid}}\cdot((\mathrm{kpC3bB}\cdot[\mathrm{pC3b}]\cdot[\mathrm{B}]-\mathrm{kmC3bB}\cdot[\mathrm{pC3bB}]))\\ & -V_{\mathrm{Fluid}}\cdot\left(\frac{\mathrm{kcat\_C3bB}\cdot[\mathrm{D}]\cdot[\mathrm{pC3bB}]}{\mathrm{Km\_C3bB}+[\mathrm{pC3bB}]}\right)\end{aligned}$$

$$\begin{aligned}\frac{\mathrm{d}\left([\mathrm{hHS}]\cdot V_{\mathrm{Fluid}}\right)}{\mathrm{d}t} =\ & -V_{\mathrm{Fluid}}\cdot((\mathrm{kpHSH}\cdot[\mathrm{hHS}]\cdot[\mathrm{H}]-\mathrm{kmHSH}\cdot[\mathrm{hHSH}]))\\ & +V_{\mathrm{Fluid}}\cdot(\mathrm{kpC3bH}\cdot[\mathrm{hC3bH}])\end{aligned}$$

$$\begin{aligned}\frac{\mathrm{d}\left([\mathrm{hHSH}]\cdot V_{\mathrm{Fluid}}\right)}{\mathrm{d}t} =\ & -V_{\mathrm{Fluid}}\cdot((\mathrm{kpC3bH}\cdot[\mathrm{hC3bBb}]\cdot[\mathrm{hHSH}]-\mathrm{kmC3bH}\cdot[\mathrm{hC3bH}]))\\ & +V_{\mathrm{Fluid}}\cdot((\mathrm{kpHSH}\cdot[\mathrm{hHS}]\cdot[\mathrm{H}]-\mathrm{kmHSH}\cdot[\mathrm{hHSH}]))\\ & -V_{\mathrm{Fluid}}\cdot((\mathrm{kpC3bH}\cdot[\mathrm{hC3b}]\cdot[\mathrm{hHSH}]-\mathrm{kmC3bH}\cdot[\mathrm{hC3bH}]))\end{aligned}$$

$$\begin{aligned}\frac{\mathrm{d}\left([\mathrm{hC3bBS}]\cdot V_{\mathrm{Fluid}}\right)}{\mathrm{d}t} =\ & -V_{\mathrm{Fluid}}\cdot(\mathrm{kpfC3b}\cdot[\mathrm{nfC3b}]\cdot[\mathrm{hC3bBS}])\\ & -V_{\mathrm{Fluid}}\cdot(\mathrm{kphC3b}\cdot[\mathrm{nhC3b}]\cdot[\mathrm{hC3bBS}])\\ & +V_{\mathrm{Fluid}}\cdot(\mathrm{kpC3bH}\cdot[\mathrm{hC3bH}])\end{aligned}$$

$$\begin{aligned}\frac{\mathrm{d}\left([\mathrm{pC3bBS}]\cdot V_{\mathrm{Fluid}}\right)}{\mathrm{d}t} =\ & -V_{\mathrm{Fluid}}\cdot(\mathrm{kppC3b}\cdot[\mathrm{npC3b}]\cdot[\mathrm{pC3bBS}])\\ & -V_{\mathrm{Fluid}}\cdot(\mathrm{kpfC3b}\cdot[\mathrm{nfC3b}]\cdot[\mathrm{pC3bBS}])\\ & +V_{\mathrm{Fluid}}\cdot(\mathrm{kpC3bH}\cdot[\mathrm{pC3bH}])\end{aligned}$$

$$\begin{aligned}\frac{\mathrm{d}\left([\mathrm{hC3bH}]\cdot V_{\mathrm{Fluid}}\right)}{\mathrm{d}t} =\ & +V_{\mathrm{Fluid}}\cdot((\mathrm{kpC3bH}\cdot[\mathrm{hC3bBb}]\cdot[\mathrm{hHSH}]-\mathrm{kmC3bH}\cdot[\mathrm{hC3bH}]))\\ & +V_{\mathrm{Fluid}}\cdot((\mathrm{kpC3bH}\cdot[\mathrm{hC3b}]\cdot[\mathrm{hHSH}]-\mathrm{kmC3bH}\cdot[\mathrm{hC3bH}]))\\ & -V_{\mathrm{Fluid}}\cdot(\mathrm{kpC3bH}\cdot[\mathrm{hC3bH}])\end{aligned}$$

$$\begin{aligned}\frac{\mathrm{d}\left([\mathrm{pPra1}]\cdot V_{\mathrm{Fluid}}\right)}{\mathrm{d}t} =\ & +V_{\mathrm{Fluid}}\cdot(\mathrm{kpC3bH}\cdot[\mathrm{pC3bH}])\\ & -V_{\mathrm{Fluid}}\cdot((\mathrm{kpPra1H}\cdot[\mathrm{pPra1}]\cdot[\mathrm{H}]-\mathrm{kmPra1H}\cdot[\mathrm{pPra1H}]))\end{aligned}$$

$$\begin{aligned}\frac{\mathrm{d}\left([\mathrm{pC3bH}]\cdot V_{\mathrm{Fluid}}\right)}{\mathrm{d}t} =\ & +V_{\mathrm{Fluid}}\cdot((\mathrm{kpC3bH}\cdot[\mathrm{pC3bBb}]\cdot[\mathrm{pPra1H}]-\mathrm{kmC3bH}\cdot[\mathrm{pC3bH}]))\\ & +V_{\mathrm{Fluid}}\cdot((\mathrm{kpC3bH}\cdot[\mathrm{pC3b}]\cdot[\mathrm{pPra1H}]-\mathrm{kmC3bH}\cdot[\mathrm{pC3bH}]))\\ & -V_{\mathrm{Fluid}}\cdot(\mathrm{kpC3bH}\cdot[\mathrm{pC3bH}])\end{aligned}$$

$$\begin{aligned}\frac{\mathrm{d}\left([\mathrm{pPra1H}]\cdot V_{\mathrm{Fluid}}\right)}{\mathrm{d}t} =\ & -V_{\mathrm{Fluid}}\cdot((\mathrm{kpC3bH}\cdot[\mathrm{pC3bBb}]\cdot[\mathrm{pPra1H}]-\mathrm{kmC3bH}\cdot[\mathrm{pC3bH}]))\\ & -V_{\mathrm{Fluid}}\cdot((\mathrm{kpC3bH}\cdot[\mathrm{pC3b}]\cdot[\mathrm{pPra1H}]-\mathrm{kmC3bH}\cdot[\mathrm{pC3bH}]))\\ & +V_{\mathrm{Fluid}}\cdot((\mathrm{kpPra1H}\cdot[\mathrm{pPra1}]\cdot[\mathrm{H}]-\mathrm{kmPra1H}\cdot[\mathrm{pPra1H}]))\end{aligned}$$
